# Supplementary material for: Human muscle-derived CLEC14A-positive cells regenerate muscle independent of PAX7
Source: Nat Commun. 2019 Dec 18;10:5776. doi: 10.1038/s41467-019-13650-z (PMC6920394; doi:10.1038/s41467-019-13650-z)
Supplement: Supplementary file 1 — Supplementary Information [file 41467_2019_13650_MOESM1_ESM.pdf]

Supplementary information to

Human muscle-derived CLEC14A-positive cells regenerate muscle independent of *PAX7*

Marg, A. et al.

Supplementary information include:

Supplementary figures 1 to 10

Supplementary tables 1 to 8

Link to online tool: https://shiny.mdc-berlin.de/humusc/

Cell lines and patient information

(Supplementary Fig. 1; Supplementary tables 1 and 2; Supplementary Movie 1)

Supplementary Table 1: Characteristics of donors

**Donors[1]**	**Number of donors**	**Age (years) Mean [range]**	**Total number of cell colonies analyzed**
PAX7null	1	5	3
Female	9	39, [16-60]	43
Male	15	45, [1-78]	57

[1]All adult patients included in this study had a normal physical examination and normal myopathological findings on muscle biopsy specimens. Complaints were cramps or myalgias. Cell colonies from eight indicated donors were performed but not included in our final analysis, because the biopsy findings revealed myopathic, inflammatory, or dystrophic features.

Supplementary Table 2: Analyses performed in human, muscle-derived, desmin-positive cell populations*

**Donor**	**Number of PAX7pos/neg CPs studied[1]**	**Immun-staining/ quantification**	**qPCR**	**Bulk RNA Seq**	**Single cell sequencing**	**Transplan tation[2]**	**Tube forming assay**	**FI[4]**
PAX7null	0/3	x	x	x	x	x	x	x
A	3/2	x	x		x +brep[3]		x	
B	2/2	x	x	x		x	x	x
C	3/0	x	x		x	x		
D	1/4	x	x					
E	0/7	x	x					
G	3/1	x	x		x +brep			
H	5/0	x	x		x +brep			
I	2/1	x	x		x		x	
J	3/1	x	x		x			
K	0/2	x	x		x +brep			
L	3/0	x	x	x				
M	0/2	x						
O	1/2	x						x
Q	4/2	x		x		x		x
R	1/3	x						x
S	3/1	x						x
T	2/2	x		x		x		x
U	2/2	x		x		x		x
W	2/1	x						x
X	0/5	x		x		x		x
Y	0/1	x						
Cc	1/2	x						

Dd	1/2	x						
Ee	1/1	x						
Hh	0/2	x						

[1]CP, cell population, determined by immunofluorescence. [2]four NOG mice were grafted/cell line unless indicated otherwise; [3]brep, biological replicate; [4]Fusion index, determined by immunofluorescence. For all experiments, only cells in early passages (<10) were used. *

Supplementary Fig. 1

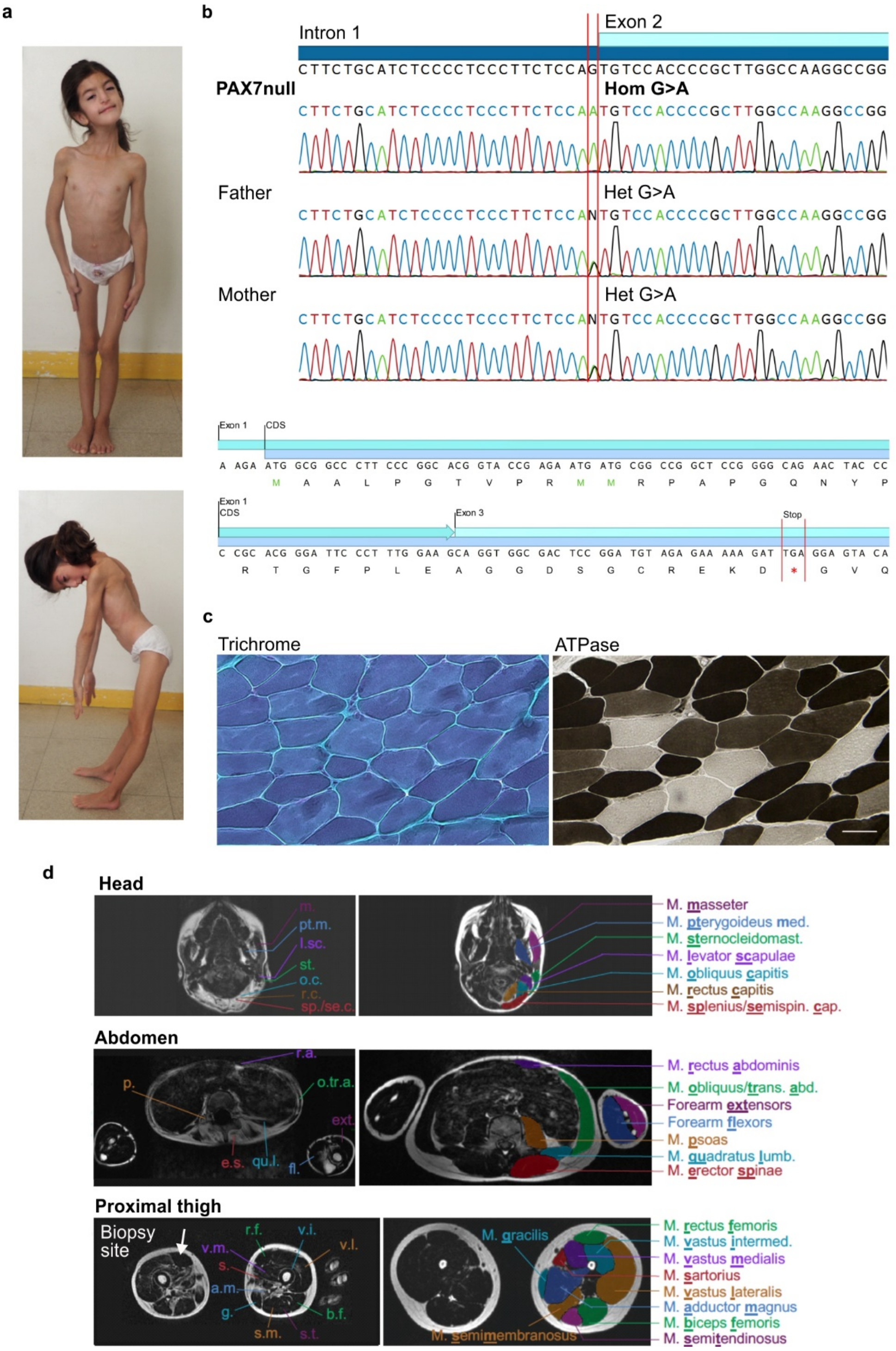

Supplementary Fig. 1 (related to Fig. 1 and Case report in the Methods-section and Supplementary Movie 1): Patient images, gene analysis, and MRI of PAX7null patient with homozygous *PAX7* c.86-1G>A mutation. **a** The patient has generalized muscle hypotrophy, a severe bilateral ptosis, mild facial dysmorphic features, hyperlordosis and a rigid spine. **b** The patient carries a homozygous splice acceptor site mutation in intron 1 of *PAX7* (c.86-1G>A). Both, father and mother, are heterozygous for the same mutation. *PAX7* c.86-1G>A leads to exon 2 skipping, resulting in a frame shift and a premature stop codon (red star) in exon 3. **c** Serial sections of muscle biopsy specimen obtained from the PAX7null patient. Muscle fiber diameters are normal for the age and without signs of necrosis, regeneration or

fibrosis. The fiber-type distribution is also normal (left panel, Gomori-Trichrome stain; right panel, ATPase, pre-incubated at pH 4.6). Scale bar: 25 µm. **d** Selected sections from whole body MRI scan. Axial images using water images of the T2 IDEAL sequence. Muscle is grey/black, adipofibrotic tissue is white. Images from head, abdomen and proximal thigh from the proband (left side) and control (right side) are chosen from anatomically comparable levels. A general muscle hypotrophy is present in the patient. The anatomy of muscles is depicted in respective colors. The M. rectus femoris is very well preserved. The site of the muscle biopsy of the M. rectus femoris is indicated by an arrow.

Supplementary Fig. 2

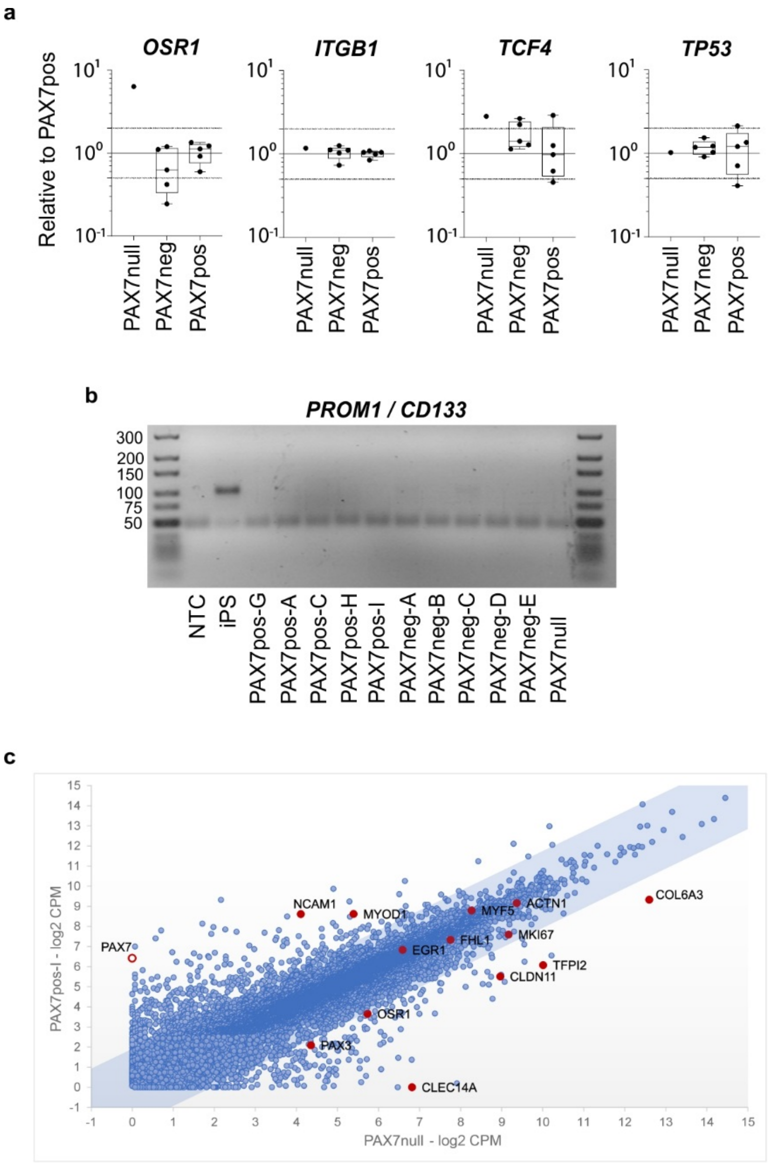

Supplementary Fig. 2 (related to Fig. 1): Expression of myogenic and interstitial cell markers. **a** qPCR analysis of additional genes expressed in human muscle derived cell colonies. All data were normalized to two reference genes and relativized to PAX7pos. The dotted line at 2fold and 0.5fold represents our threshold for considering a gene differentially expressed. **b** qRT-PCR analysis of PROM1 (CD133) expression in human muscle-derived cells. An induced pluripotent stem (iPS) cell line generated by us was used as positive control. NTC = No Template Control. Cell lines are described in Supplementary Table 2. **c** Bulk RNASeq**.** Scatter-plot of the normalized count data from PAX7null and PAX7pos-I. PAX7null cells have zero read counts for PAX7 (non-filled circle). Information on cells in SupplementaryTable 2.

Supplementary Fig. 3

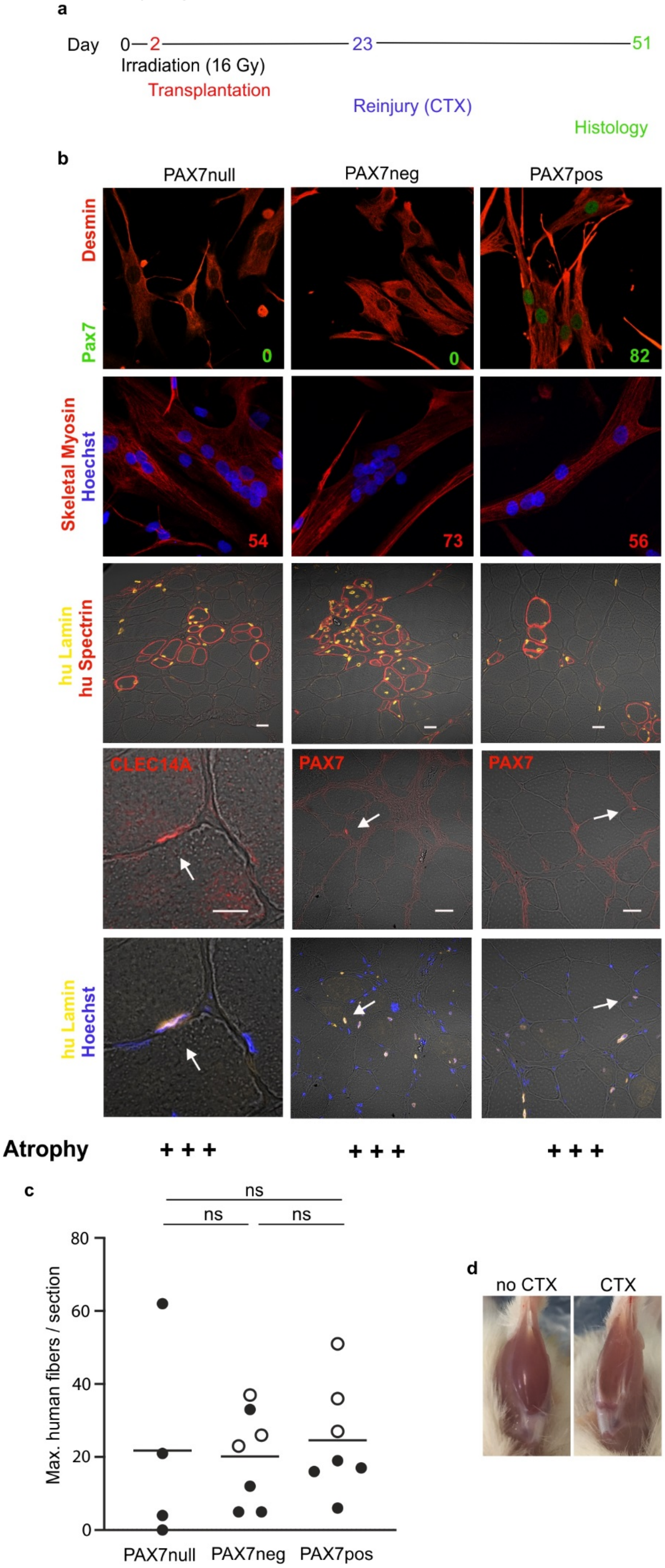

Supplementary Fig. 3 Muscle regeneration after reinjury. **a** Time line of the reinjury experiment. **b** Panels in line 1 and 2: Characterization of PAX7null, PAX7neg and PAX7pos cells before transplantation into *NOG* mice. Green: % of PAX7pos cells; red: fusion index. Panels in line 3-5: Characterisation of muscles of *NOG* mice after transplantation + reinjury. Panels in line 3 display immunofluorescent analyses using antibodies against human spectrin (red) and human lamin A/C (yellow). Scale bars: 20 µm. The left panel in line 4 shows a CLEC14A positive cell (arrow) in a satellite cell position after transplantation of PAX7null cells. The middle and right panel show PAX7+ cells observed after transplantation of PAX7neg and PAX7pos cells (line 4). Scale bar, left: 10 µm. Scale bars, middle and right: 20 µm. Panels in line 5 display the counterstains, i.e. human lamin A/C (yellow) and Hoechst (blue). **c** Quantification of transplantation experiments shown in b (analysis performed as reported for Fig.3). Each dot represents a grafted mouse. Open circles indicate that PAX7+ cells of human origin were identified in the graft. The mean values are indicated by the line. Statistical differences between the groups were analyzed by two-tailed *t* tests, $P > 0.05$ = n.s. (not significant). **d** All grafted muscles were severely atrophic following irradiation and reinjury.

Supplementary Fig. 4

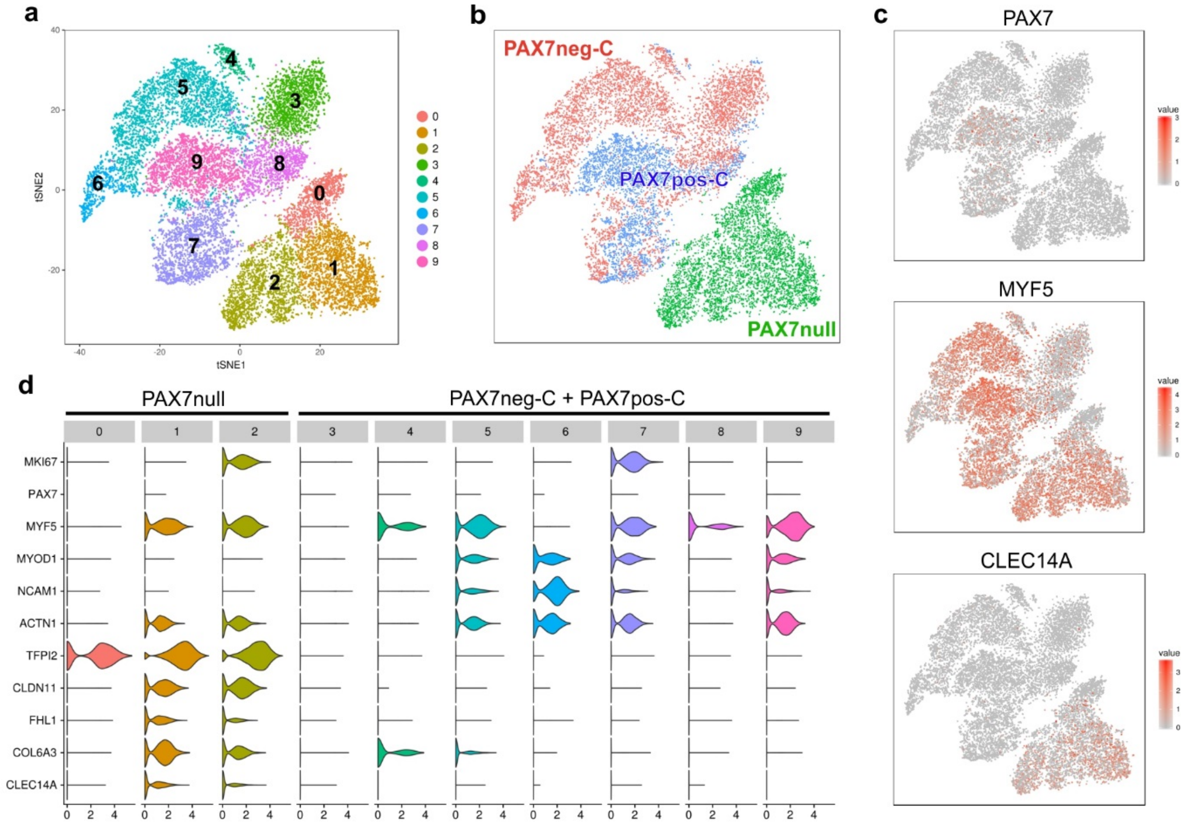

Supplementary Fig. 4 (related to Fig. 2): Single-cell transcriptomics reveals differences between PAX7null, PAX7pos, and PAX7neg myogenic cell colonies. **a-c** Two-dimensional tSNE plots of single cell data obtained from PAX7null as well as PAX7pos and PAX7neg colonies from donor C. Each dot represents a cell. **a** tSNE plots colored by PAX7 status. **b** tSNE plots colored by cell population. **c** Normalized gene expression levels of selected marker genes (red: high, grey: low). **d** Violin plots indicating gene expression levels and distributions of selected genes within cell clusters corresponding to panel a.

Supplementary Fig. 5

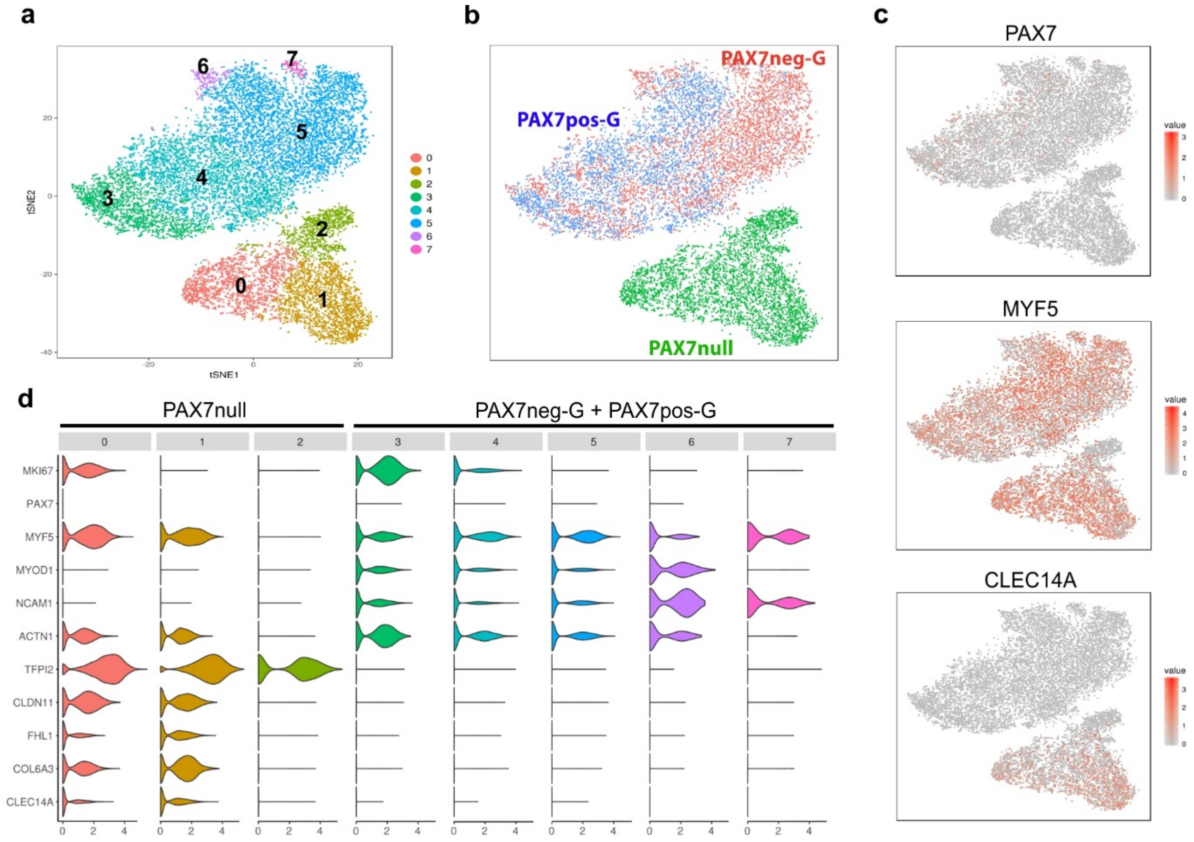

Supplementary Fig. 5 (related to Fig. 2): Single-cell transcriptomics reveals differences between PAX7null, Pax7pos and PAX7-neg myogenic cell colonies. Here**:** PAX7-deficient donor (PAX7null) compared to PAX7pos/PAX7neg colonies obtained from donor G. For details, see Supplementary Fig. 5.

Supplementary Fig. 6

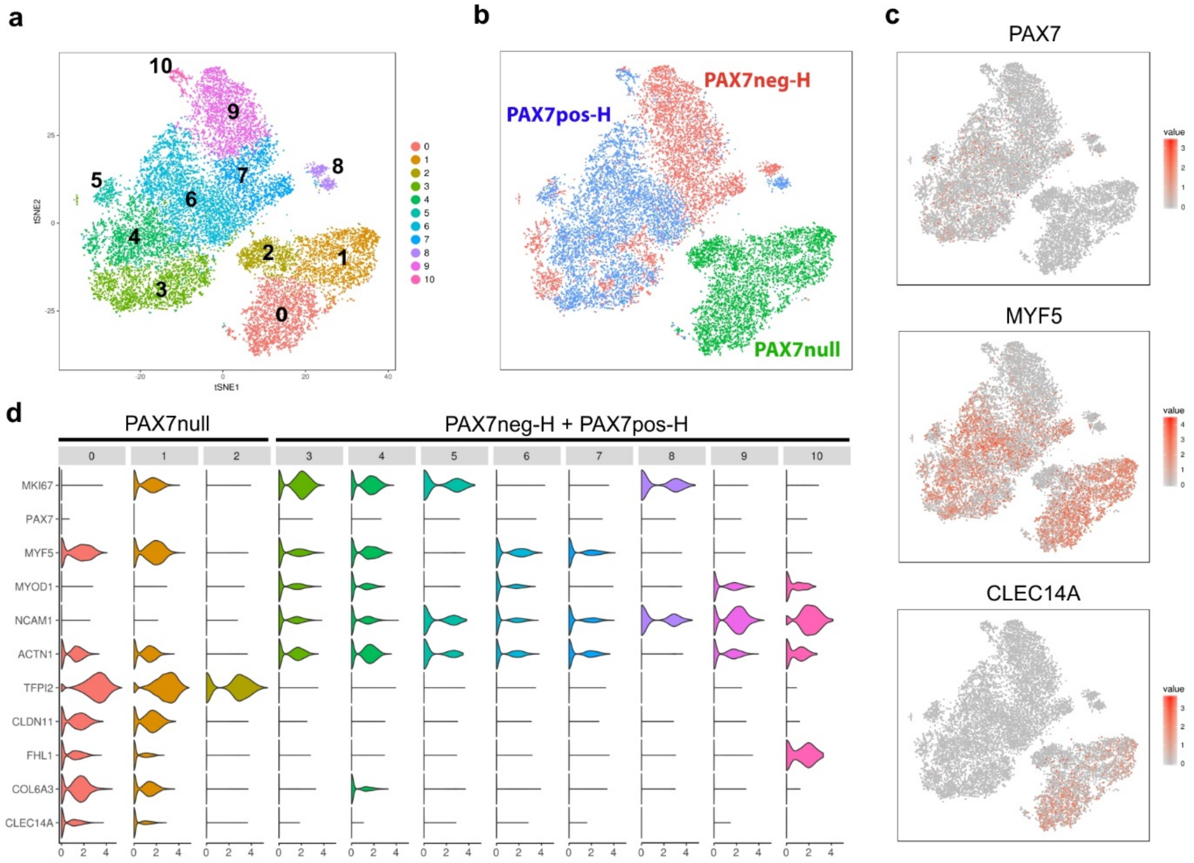

Supplementary Fig. 6 (related to Fig. 2): Single-cell transcriptomics reveals differences between PAX7null, PAX7pos and PAX7neg myogenic cell colonies. Here**:** PAX7-deficient donor (PAX7null) compared to PAX7pos/PAX7neg colonies obtained from donor H. For details, see Supplementary Fig. 5.

Supplementary Fig. 7

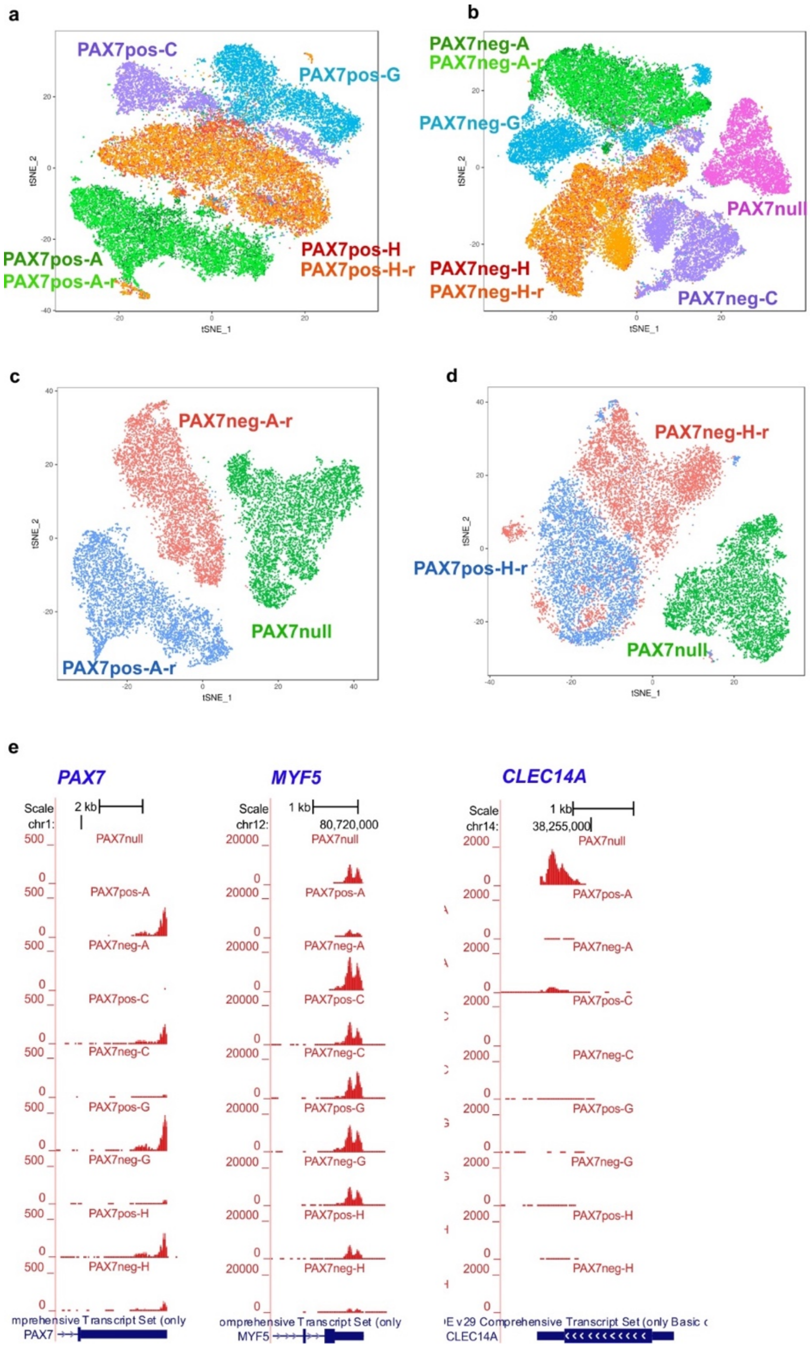

Supplementary Fig. 7 (related to Fig. 2): Extent of transcriptional heterogeneity in different human myogenic cell colonies. **a, b** Two-dimensional tSNE representation of single cell transcriptomic data obtained from the different cell colonies used in this study. Each dot represents a cell. Biological replicates (marked by "-r") cluster together (shown are colonies from donor A and H as an example). **a** PAX7pos colonies. **b** PAX7neg colonies. **c** Data obtained from biological replicates of PAX7pos and PAX7neg colonies from donor A compared to PAX7null cells (corresponding to Fig. 3b). **d** Data obtained from biological replicates of PAX7pos and PAX7neg colonies from donor H compared to PAX7null cells (corresponding to Supplementary Fig. 5b). **e** Genome browser tracks displaying sequencing reads mapped to the 3' ends of the indicated genes. Arrowheads indicate direction of transcription. Blue lines indicate intron (thin stippled line), coding region (thick line) or 3'UTR (medium line). *PAX7* expression was not detected in PAX7null cells, while *CLEC14A* expression was high. *MYF5* expression was detected in all cell populations.

Supplementary Fig. 8

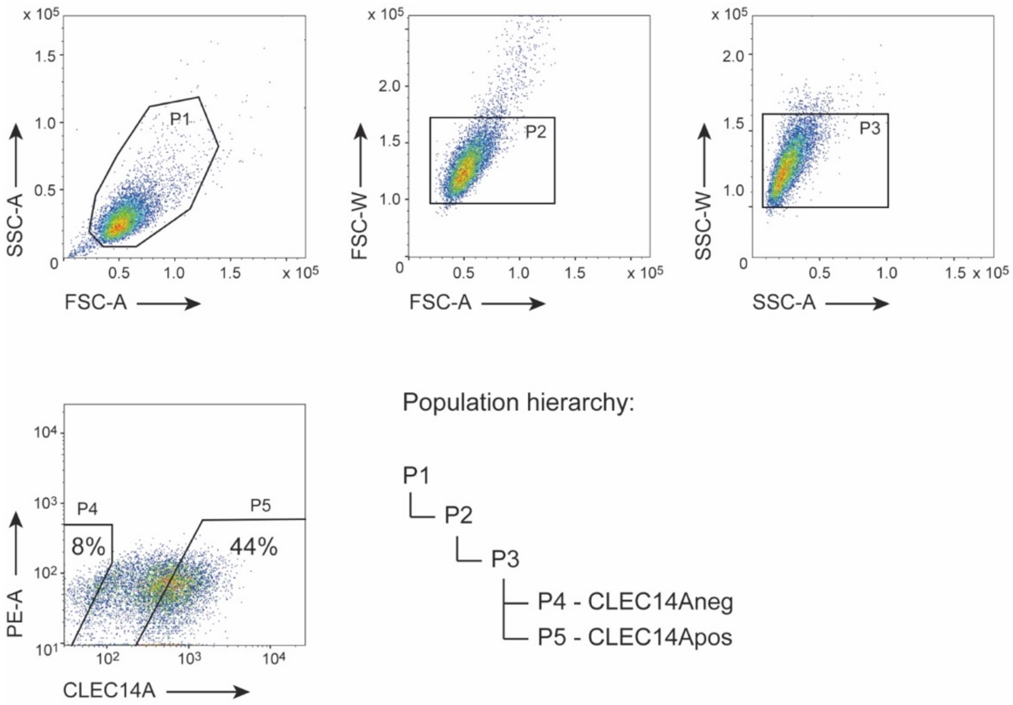

Supplementary Fig. 8 Gating strategy for FACS sorting of CLEC14A positive and negative primary human myoblasts. Representative plots of a primary human myoblast sample stained with an anti-CLEC14A antibody and an Alexa Fluor® 488-conjugated secondary antibody. Gates were defined to select for the viable cell population (P1) and for doublet exclusion (P2, P3). CLEC14Aneg (P4) and CLEC14Apos (P5) events were separated according to their green fluorescence intensity using stringent gates to ensure a high purity sorting. The experiment was performed three times.

Supplementary Fig. 9

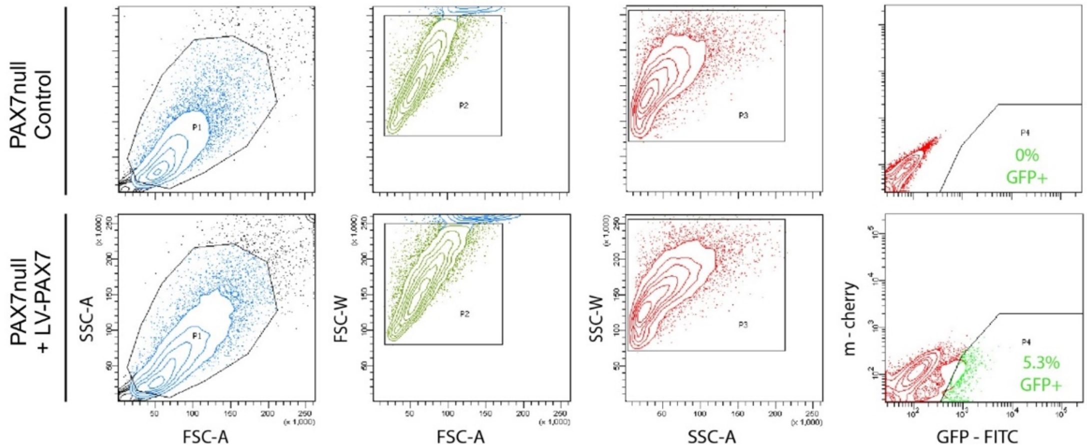

Supplementary Fig. 9 (related to Fig. 4): Selection of PAX7null cells following transduction with a lentiviral expression vector for PAX7. We used FACS-sorting to enrich for PAX7null cells transduced with a lentiviral vector carrying a constitutively expressed GFP reporter and a doxycycline-inducible PAX7 expression cassette. Dead cells, debris and cell doublets were excluded. The GFP positive fraction was gated using the FITC channel. Laser interference was avoided by plotting the FITC signal against m-Cherry. A stringent setting of the GFP – FITC gate was used to avoid false positive events.

Supplementary Fig. 10

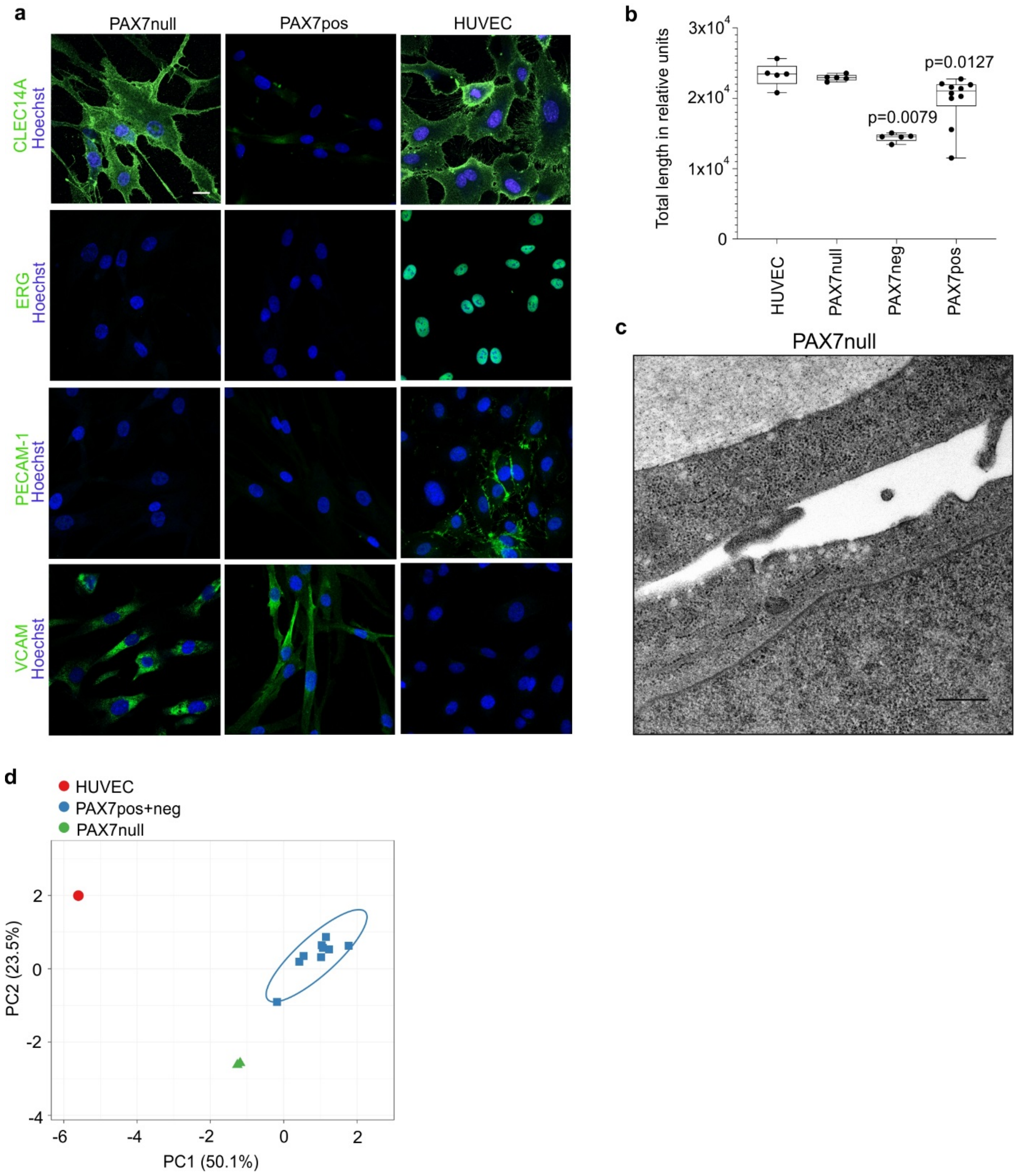

Supplementary Fig. 10 Analyses of endothelial markers expressed in human muscle derived cell colonies. **a** Staining of PAX7null, PAX7pos and HUVEC cells for CLEC14A, ERG, PECAM-1 and VCAM1 (green). Cells were counterstained with Hoechst (blue). Scale bar: 20 µm. **b** ImageJ Angiogenesis Analyzer plugin determined the sum of the lengths of segments and branches of tubes formed by the cultured cells. Mann-Whitney test was used to test for signifcance between tubes formed by HUVEC cells and PAX7pos or PAX7null cells. **c** Ultrathin sections of epon embedded PAX7null tubes formed after 12h culture on Matrigel®. Multiple caveolae are depicted. Scale bar: 500 nm. **d** PCA plot from bulk RNAseq of ten PAX7pos/PAX7neg and PAX7null cell colonies. HUVEC bulk RNAseq data were download from public GEO database (SRR7549223, SRR7549223, SRR7549225). Information about the cell lines is provided in Supplementary Table 2.

Supplementary table 3: Potentially pathogenic variants annotated by the MutationTaster2 software in the autozygous genomic regions of the index patient and their allele frequency in the gnomAD database (https://gnomad.broadinstitute.org/). (related to Fig. 1 and Supplementary Fig. 1)

Gene symbol	Protein name	Transcript	Chr	Pos	Ref	Alt	AAE	Zygostiy	Coverage	gnomAD			
										het	homo	total number of alleles	allele frequency
**Autozygous region**													
EPHA2	EPH receptor A2	ENST00000358432	1	16,475,172	C	T	p.R175H	homo	81	10	0	248,898	4.02E-05
PAX7	Paired box 7	ENST00000420770	1	18,960,796	G	A	splice site change	homo	62	0	0	247,812	0.00E+00
TRABD2B	TraB domain containing 2B	ENST00000606738	1	48,260,410	G	C	p.T279S	homo	103	88	0	165,756	5.31E-04
DSP	Desmoplakin	ENST00000379802	6	7,576,619	G	A	p.R908H	homo	94	302	1	282,726	1.07E-03
TRPM3	Transient receptor potential cation channel subfamily M member 3	ENST00000358082	9	73,225,639	C	G	p.E701D	homo	134	1523	9	282,832	5.38E-03
MBD6	Methyl-CpG binding domain protein 6	ENST00000355673	12	57,922,485	C	T	p.R953C	homo	255	209	0	282,838	7.39E-04
RPTOR	Regulatory associated protein of MTOR complex 1	ENST00000306801	17	78,935,275	T	C	no AA change	homo	316	529	2	187,348	2.82E-03
FN3K	Fructosamine 3 kinase	ENST00000300784	17	80,708,587	T	A	p.Y296N	homo	192	76	2	250,934	3.03E-04
**Panel 1**													
none													
**Panel 2**													
none													
**Panel 3**													
GFPT1	Glutamine-fructose-6-phosphate transaminase 1	ENST00000361060	2	69,586,410	T	C	p.K133R	het	100	1	0	251,150	3.98E-06
PLEC	Plectin	ENST00000356346	8	144,990,701	C	T	p.A4416T	het	89	1	0	241,904	4.13E-06
MUSK	Muscle associated receptor tyrosine kinase	ENST00000189978	9	113,562,589	T	C	p.V644A	het	232	807	4	276,616	2.92E-03

Chr, chromosome; **Pos**, physical position; **Ref**, reference allele; **Alt**, alternative allele; **AAE**; amino acid exchange; **het**, heterozygous; **homo**, homozygous.

Supplementary table 4: Primer sequences for sanger sequencing (related to Fig. 1 and Supplementary Fig 1)

Primer target	Orientation	Sequence	Accession-No
PAX7 gDNA exon 1	Fwd 5'UTR	CCCCTCGCTTTTCCATTT	NG_023262.1
	Rev Intron 1	CCCAGAAGAGAACGGGAT	
PAX7 gDNA exon 2	Fwd Intron 1	GCTGTGACTCCTCTATCCAT	
	Rev Intron 2	ACCTCACTTAGCTCCATCTC	
PAX7 gDNA exon 3	Fwd Intron 2	AGGATAAAGCCAATCTCTTG	
	Rev Intron 3	TCCTCCCAACTTCTCAGTAA	
PAX7 gDNA exon 4	Fwd Intron 3	TGTGTGTGGAAGAGGGATGA	
	Rev Intron 4	CCCGCAGCATGAGAGTTT	
PAX7 gDNA exon 5	Fwd Intron 4	GGAGGAGTCTGATCGAAGTG	
	Rev Intron 5	CTGCCAACTAACTCTGCATC	
PAX7 gDNA exon 6	Fwd Intron 5	ATGCTGGCTGGATCAGAGG	
	Rev Intron 6	TTCAAGGGCAGGATTGCATCT	
PAX7 gDNA exon 7	Fwd Intron 6	TGTCTCATCCCAGGTAGCAT	
	Rev Intron 7	GAAAAGCCAAGAGCACAGGA	
PAX7 gDNA exon 8	Fwd Intron 7	TCAAGAGAAACACCGAAGACC	
	Rev Intron 8	ACCCTGACACCACCTTGTAG	
PAX7 gDNA exon 9	Fwd Intron 8	TGATTGGAGAGGAGGTGAGAG	
	Rev Exon 9	GAAGCTCAGGGGTCAGTTAG	
PAX7 gDNA ex9 - 3UTR	Fwd Exon 9	GACTCACAGCCACTTTCC	
	Rev 3'UTR	TAGGCATTCACTAGAAACCTC	
PAX7 mRNA exon1 - exon 5	Fwd Exon 1	ATCGCAGCAGGGGTGAA	NM_002584.2
	Rev Exon 5	TCCTCGCGGGTGTATATGTC	
PAX7 mRNA exon 1 - exon 3	Fwd Exon 1	ATCGCAGCAGGGGTGAA	
	Rev Exon 3	ATGCCTGGGTTTTCCCTCTT	
PAX7 mRNAexon5 - exon 8	Fwd Exon 5	AGAGGACCCACTACCCAGA	
	Rev Exon 8	TATTTCTGTTGGAGCCATAGTACG	

Supplementary table 5: Antibodies, source and concentration (related to Fig. 1,3,4, Supplementary Fig. 3)

Antibody	Catalog number	Working concentration
CD31 (PECAM1)	ab28364, Abcam	1: 20
CD56 (NCAM)	130-090-955, Miltenyi Biotec	1: 10
Hu CLEC14A	PA5-47677, Thermo Fisher Scientific	1: 40; w/o permeabilization
Desmin	ab15200, Abcam	1: 2.000
ERG	ab92513, Abcam	1: 100
Hu Lamin A+C	ab108595, Abcam	1: 4.000
Laminin, DyLight 488	PA5-22901, Thermo Fisher Scientific	1: 100
MYF-5 (C20)	sc-302, Santa Cruz Biotechnology	1: 2.000
PAX3 (F-2)	sc-376204, Santa Cruz Biotechnology	1: 100
PAX7	sc-81648, Santa Cruz Biotechnology	cells: 1: 200, sections: 1: 100
Skeletal Myosin (FAST), Clone MY-32	M 4276, Sigma-Aldrich	1: 200
Hu Spectrin	NCL-SPEC1, Leica Biosystems	1: 100
Syndecan-4 (H140)	sc-15350, Santa Cruz Biotechnology	1: 25
VCAM1	ab134047, Abcam	1: 300

Supplementary table 6: Primer sequences for quantitative PCR (related to Fig. 1)

Primer target	Orientation	Sequence	Accession-No
CLEC14A	Fwd Exon 1	GCCAAGGAAGGAGTCTATGG	NM_175060.2
	Rev Exon 1	CCGACTTTCACCCCATTGTT	
Cyclophilin	Fwd Exon 1	CGCCGAGGAAAACCGTGTACTATT	NM_021130.3
	Rev Exon 1/2	GACCTTGTCTGCAAACAGCTCAAAG	
GAPDH	Fwd Exon 2	GAAGGTGAAGGTCGGAGTC	NM_002046
	Rev Exon 5	GAAGATGGTGATGGGATTTC	
ITGB1/CD29	Fwd Ex 12/13	TGTGGAGGAAATGGTGTTTGC	NM_002211.3
	Rev Exon 13	ATCTGTCCGTTGCTGGCTTC	
MYOD1	Fwd Exon 2	GCGGAACTGCTACGAA	NM_002478
	Rev Exon 3	AGATGCGCTCCACGAT	
NCAM1	Fwd Exon 6/7	GGACAAAGGATGGGGAACAGATAG	NM_001242607.1
	Rev Exon 7	CTCAGCCTCGTCGTTCTTATC	
OSR1	Fwd Exon 1	TTCCCCAGTCCCCTTCA	NM_145260.2
	Rev Exon 2	CAAGGTTTTGCTGCCCATTT	
PAX3	Fwd Exon 6/7	CAAGCTGTGTCAGATCCC	NM_181457.3
	Rev Exon 7	AGGAATCGTGCTTTGGTGTA	
PAX7	Fwd Exon 4/5	TGGGCGACAAAGGGAA	NM_002584.2

	Rev Exon 5	GGTAGTGGGTCCTCTCAAA	
PDGFRa	Fwd Exon 5/6	GGAGAAGTGAAAGGCAAAGG	NM_006206.5
	Rev Exon 6	GGCAGCACATTCGTAATCTC	
PW1/PEG3	Fwd Exon 8/9	GCTTGCTGAAGACGATG	NM_001146187.1
	Rev Exon 10	ATCACTCCGTGGGAAGATT	
TCF4	Fwd Ex 10/11	ACGTTTGAGCTATCCATCACA	NM_001083962.1
	Rev Exon 11	AGGAGGCGTACAGGAAGAG	
TP53	Fwd Exon 4	CATTCTGGGACAGCCAAGTC	NM_000546.5
	Rev Exon 5	CTGTGACTGCTTGTAGATGG	
PROM1/CD133	Fwd Exon 1	GGTACAGCCGCGTGATTT	NM_006017.2
	Rev Exon 2/3	AGATTACAGTTTCTGGCTTGTC	

Supplementary table 7 (related to Fig. 2): Quality assessment of single cell sequencing results

Sample	Cells (median)	Genes (median)	umis (median)	Reads (median)
PAX7neg-A	4791	1392	2508	4897
PAX7neg-A_r2[1]	4648	1467	2496	4374
PAX7neg-C	6576	1218	2172	5014
PAX7neg-G	4956	921	1401	3658
PAX7neg-H	5333	1147	1944	3643
PAX7neg-H_r2	5605	766	1102	1967
PAX7null	4950	1708	3534	7300
PAX7pos-A	3777	1039	1676	3782
PAX7pos-A_r2	4492	1320	2165	4450
PAX7pos-C	3726	1704	3484	6338
PAX7pos-G	5105	1358	2351	7728
PAX7pos-H	6833	1358	2339	4138
PAX7pos-H_r2	5244	1590	2793	4502
**Average (mean)**	**5080**	**1307**	**2305**	**4753**
Total	66036			

Supplementary Table 8: Quality of bulk RNA-seq and alignment report (against GRCh38) Raw data files and read counts can be accessed via GeoOmnibus bioproject PRJNA481958.

Sample	Total read number (for mapping)	Mapped pairs (%)	Mapped to genes (%)
PAX7null	68.829.772	89,26	94,68
PAX7null-rep	83.395.158	91,91	95,53
PAX7neg-B	63.717.280	92,68	95,72
PAX7pos-I	70.957.122	91,56	95,19
PAX7pos-Q	83.324.390	92,69	96,34
PAX7neg-Q	91.848.654	92,15	97,55
PAX7pos-T	94.796.892	92,11	96,13
PAX7neg-T	87.313.444	92,26	95,96
CLEC14Apos-U	86.973.134	93,39	96,32
CLEC14Aneg-U	88.264.020	90,91	96,52
CLEC14Apos-X	88.545.822	88,67	96,15
