# Supplementary material for: Human muscle-derived CLEC14A-positive cells regenerate muscle independent of PAX7
Source: Nat Commun. 2019 Dec 18;10:5776. doi: 10.1038/s41467-019-13650-z (PMC6920394; doi:10.1038/s41467-019-13650-z)
Supplement: Supplementary file 5 — Reporting Summary [file 41467_2019_13650_MOESM5_ESM.pdf]

nature research

Corresponding author(s): Simone Spuler

Reporting Summary

Nature Research wishes to improve the reproducibility of the work that we publish. This form provides structure for consistency and transparency in reporting. For further information on Nature Research policies, see Authors & Referees and the Editorial Policy Checklist.

Statistical parameters

When statistical analyses are reported, confirm that the following items are present in the relevant location (e.g. figure legend, table legend, main text, or Methods section).

n/a	Confirmed	
☐	☒	The exact sample size (*n*) for each experimental group/condition, given as a discrete number and unit of measurement
☐	☒	An indication of whether measurements were taken from distinct samples or whether the same sample was measured repeatedly
☐	☒	The statistical test(s) used AND whether they are one- or two-sided<br>*Only common tests should be described solely by name; describe more complex techniques in the Methods section.*
☒	☐	A description of all covariates tested
☒	☐	A description of any assumptions or corrections, such as tests of normality and adjustment for multiple comparisons
☒	☐	A full description of the statistics including central tendency (e.g. means) or other basic estimates (e.g. regression coefficient) AND variation (e.g. standard deviation) or associated estimates of uncertainty (e.g. confidence intervals)
☒	☐	For null hypothesis testing, the test statistic (e.g. *F*, *t*, *r*) with confidence intervals, effect sizes, degrees of freedom and *P* value noted<br>*Give P values as exact values whenever suitable.*
☒	☐	For Bayesian analysis, information on the choice of priors and Markov chain Monte Carlo settings
☐	☒	For hierarchical and complex designs, identification of the appropriate level for tests and full reporting of outcomes
☒	☐	Estimates of effect sizes (e.g. Cohen's *d*, Pearson's *r*), indicating how they were calculated
☒	☐	Clearly defined error bars<br>*State explicitly what error bars represent (e.g. SD, SE, CI)*

Our web collection on statistics for biologists may be useful.

Software and code

Policy information about availability of computer code

Data collection	qPCR data were collected with MxPro software (v 4.1; Stratagene).<br>Microscopic images, except for tube formation images, were collected using ZEN 2.3 (Carl Zeiss Microscopy GmbH).<br>iTEM software (Emsis GmbH, Germany) for electron microscopy.<br>EVOS FL (Thermo Fisher) for tube formation images.<br>BD FACS Diva for FACS sorting (BD Bioscience).
Data analysis	CLC Genomics Workbench v12.0 (Qiagen, Germany), CLUSTVis for bulk RNA sequencing data analysis.<br>CLC Genomics Workbench v9.5 (Qiagen, Germany) for sanger sequencing analysis.<br>Drop-seq Toolkit v.1.13, Seurat v.2.3, and STAR v2.4.j for single cell transcriptomics.<br>EXCEL2016, GraphPad Prism v8 for calculation, statistical analyses, and plotting graphs.<br>BWA-MEM v.0.7.1, GATK v3.8, and MutationTaster2 for analysing whole exome sequencing data.<br>ImageJ Angiogenesis Analyzer for quantifying tube formation.

For manuscripts utilizing custom algorithms or software that are central to the research but not yet described in published literature, software must be made available to editors/reviewers upon request. We strongly encourage code deposition in a community repository (e.g. GitHub). See the Nature Research guidelines for submitting code & software for further information.

Data

Policy information about availability of data

All manuscripts must include a data availability statement. This statement should provide the following information, where applicable:

- Accession codes, unique identifiers, or web links for publicly available datasets
- A list of figures that have associated raw data
- A description of any restrictions on data availability

Bulk RNA-seq and Single-cell data are restored at NCBI GEO Omnibus via GSE117382. The data are not open to the public yet. The following token has been created to allow reviewing of record GSE117382 at GEO Omnibus while it remains in private status: mfmdsiievnurlsz. This is for reviewing process only.

Field-specific reporting

Please select the best fit for your research. If you are not sure, read the appropriate sections before making your selection.

☒ Life sciences ☐ Behavioural & social sciences ☐ Ecological, evolutionary & environmental sciences

For a reference copy of the document with all sections, see nature.com/authors/policies/ReportingSummary-flat.pdf

Life sciences study design

All studies must disclose on these points even when the disclosure is negative.

Sample size	Sample size was determined by availability of patient material.
Data exclusions	qPCR amplification data have been excluded if replicates did not meet the MIQE criteria. The experiment was repeated.
Replication	We used biological replicates from different human donors. The human PAX7 deficient patient is unique.
Randomization	Randomization was not required in our investigation.
Blinding	Scientists were blinded for quantification of PAX7 content of human muscle stem cells and transplantation efficiency.

Reporting for specific materials, systems and methods

Materials & experimental systems

n/a	Involved in the study	
☐	☒	Unique biological materials
☐	☒	Antibodies
☐	☒	Eukaryotic cell lines
☒	☐	Palaeontology
☐	☒	Animals and other organisms
☐	☒	Human research participants

Methods

n/a	Involved in the study	
☒	☐	ChIP-seq
☐	☒	Flow cytometry
☒	☐	MRI-based neuroimaging

Unique biological materials

Policy information about availability of materials

Obtaining unique materials	PAX7 deficient patient (4y) is a patient from our outpatient clinic for muscular diseases and carries an unique PAX7 splice site mutation. Human muscle biopsy material is restricted regarding availability and quantity, also for ethical reasons.

Antibodies

Antibodies used

Antibody, Catalog number, Working concentration
CD31 (PECAM1), ab28364, Abcam, Lot: GR3247741-1, 1: 20
CD56 (NCAM), 130-090-955, Miltenyi Biotec, Lot: 5190110029, 1: 10

CLEC14A, PA5-47677, Thermo Fisher Scientific, Lot: TB2523542A, 1: 40; w/o permeabilization
Desmin, ab15200, Abcam, Lot: GR2850024-3, 1: 2.000
ERG, ab92513, Abcam, Lot: GR3220764-2, 1: 100
Hu Lamin A+C, ab-108595, Abcam, Lot: GR192728-4, 1: 4.000
Laminin-DyLight 488, PA5-22901, Thermo Fisher Scientific , Lot: TK2665370D, 1: 100
MYF-5, (C-20) sc-302, Santa Cruz Biotechnology, Lot: D1515, 1: 2.000
PAX3 (F-2), sc-376204, Santa Cruz Biotechnology, Lot: D1516, 1: 100
PAX7, sc-81648, Santa Cruz Biotechnology, Lot: D2417, cells: 1: 200, sections: 1: 100
Skeletal Myosin (FAST), Clone MY-32 M 4276, Sigma-Aldrich, Lot: 096M4862V, 1: 200
Hu Spectrin NCL-SPEC1, Leica Biosystems, Lot: 6060454, 1: 100
Syndecan-4 (H140), sc-15350, Santa Cruz Biotechnology, Lot: C1207, 1: 25
VCAM1, ab134047, Abcam, Lot: GR257919-54, 1: 300

Validation

Antibodies were only used if the manufacturer provided sufficient information about specificity and purity. We performed appropriate controls, i.e. omission of 1st ab, isotype control, ab – dilution before ab was used in experiments.

Eukaryotic cell lines

Policy information about cell lines

Cell line source(s)

State the source of each cell line used.

Authentication

Describe the authentication procedures for each cell line used OR declare that none of the cell lines used were authenticated.

Mycoplasma contamination

Confirm that all cell lines tested negative for mycoplasma contamination OR describe the results of the testing for mycoplasma contamination OR declare that the cell lines were not tested for mycoplasma contamination.

Commonly misidentified lines
(See ICLAC register)

Name any commonly misidentified cell lines used in the study and provide a rationale for their use.

Animals and other organisms

Policy information about studies involving animals; ARRIVE guidelines recommended for reporting animal research

Laboratory animals

5-7-week old male NOD.Cg-PrkdcscidIl2rgtm1Sug/JicTac (NOG) mice were purchased from Taconic Biosciences.

Wild animals

The study did not involve wild animals.

Field-collected samples

The study did not involve field-collected animals or samples.

Human research participants

Policy information about studies involving human research participants

Population characteristics

81 diagnostic muscle biopsy specimens, 10 female (age 4 - 83 years), 16 male (age 1 - 78 years). All patients were recruited through the University Outpatient Clinic for Muscle Disorders at the Charité Universitätsmedizin Berlin. Muscle stem cell population data of 65/81 donors are only included in Fig. 4c. Experimental usage of cell colonies of 26 donors are in detail described in Supplementary Table 2.

Recruitment

All donors and the PAX7 deficient patient are patients of our University outpatient clinic for muscle disorders.

Flow Cytometry

Plots

Confirm that:

- [x] The axis labels state the marker and fluorochrome used (e.g. CD4-FITC).
- [x] The axis scales are clearly visible. Include numbers along axes only for bottom left plot of group (a 'group' is an analysis of identical markers).
- [x] All plots are contour plots with outliers or pseudocolor plots.
- [x] A numerical value for number of cells or percentage (with statistics) is provided.

Methodology

Sample preparation

Primary myoblasts were thawed from liquid nitrogen and kept for 2 passages in standard muscle medium. 4x10^6 PAX7null cells (5.2x10^3/cm2) were infected with a PAX7-GFP-Lentivirus with an MOI of 7.8 and FACS-sorted 4 days after infection using the

	GFP reporter.
Instrument	FACSAria™ Fusion, BD Biosciences
Software	BD FACS Diva software was used to record and analyse the cell sorting.
Cell population abundance	After sorting we found 5.3% GFP positive cells that were expanded and used for the experiments.
Gating strategy	We used the FSC/SSC plot to define the viable cell population and to exclude debris. FSC-W/FSC-A and SSC-W/SSC-A plots were used for doublet exclusion. The GFP positive fraction was gated using the FITC channel. Laser interference was avoided by plotting the FITC signal against m-cherry. The GFP - FITC gate was set stringent to avoid false positive cells from the GFP negative cell population.

☒ Tick this box to confirm that a figure exemplifying the gating strategy is provided in the Supplementary Information.
