# Supplementary material for: Human muscle-derived CLEC14A-positive cells regenerate muscle independent of PAX7
Source: Nat Commun. 2019 Dec 18;10:5776. doi: 10.1038/s41467-019-13650-z (PMC6920394; doi:10.1038/s41467-019-13650-z)
Supplement: Supplementary file 6 — Description of Additional Supplementary Files [file 41467_2019_13650_MOESM6_ESM.pdf]

Title: Supplementary Data 1
Description: Read counts and expression values from bulk RNA-seq data. Raw files can be accessed via GeoOmnibus number SRA accession: PRJNA481958

Title: Supplementary Data 2
Description: A full table listing discriminating genes and quantification measures between the populations is provided as Supplementary Data File 2 in a separate Excel file.

Title: Supplementary Movie 1
Description: Summary of clinical findings in PAX7null patient
